# The concept of Narcissistic Personality Disorder–Three levels of analysis for interdisciplinary integration

**DOI:** 10.3389/fpsyt.2022.989171

**Published:** 2022-11-16

**Authors:** Kerrin A. Jacobs

**Affiliations:** ^1^Department of Philosophy and Ethics, Faculty of Humanities and Human Sciences, University of Hokkaido, Sapporo, Japan; ^2^Center for Human Nature, Artificial Intelligence, and Neuroscience (CHAIN), University of Hokkaido, Sapporo, Japan

**Keywords:** Narcissistic Personality Disorder, empathy, psychoanalysis, phenomenology, reductionism, integration

## Abstract

In this paper, I distinguish three different levels for describing, and three corresponding ways for understanding, deficient empathy as the core of NPD (Narcissistic Personality Disorder). On the macro level, deficient empathy can be explained as disturbed interpersonal functioning, and is understood as lack of *re*cognition. On the meso-level, deficient empathy can be described as psychic disintegration, and can be understood specifically in its dissocial aspects. Psychic disintegration in NPD correlates with somatic changes, i.e., dysfunctional affective empathy and mind-reading on the micro level of description, which is the third level. The “core-deficit-model of NPD” that I outline, while not rejecting reductionist approaches outright, argues in favor of integrating (top-down/bottom-up) functionalist descriptions of empathy into a wider conceptual framework of bio-psycho-social functioning. The “core-deficit-model of NPD” is interdisciplinary, can bypass monodisciplinary skepticism, and removes purported barriers between explaining and understanding the “lack” of empathy as the core of pathological narcissism.

## Introduction

A scientific explanation of an empathy deficit in terms of biological dysfunctions is usually considered as a bottom up approach, while a hermeneutic understanding of the deficit as a (harmful) impairment of normal interpersonal functioning is usually considered as top down. In this paper, I will first look at well-known symptoms associated with conditions of trait narcissism and pathological narcissism. It turns out that an important symptom is an diminished ability to empathically engage with other persons. This feature stands in need of deeper explanation. If one adopts a gradualist view of psychic capacities, persons with NPD apparently do not lack empathy altogether, but rather they show a more or less reduced propensity of empathic concern. Instead of empathic concern, persons with severe NPD often develop a rather objectifying view of other persons. This objectifying mode of affective detachment is specific for narcissistic cognition (1).^1^

If one wants to bridge the conceptual gap between the world of neuroscientific naturalistic explanations of mental processing in persons with NPD, and hermeneutic explanations of their ways of interacting with others, then bidirectional causal relations between the intrapsychic realm and the interpsychic realm of functioning can serve as the basis for coordinating heterogeneous functional descriptions at different levels of analysis. I will address some expectable methodological doubts about such an integrative attempt later on. The conclusion I want to substantiate is that deficient empathy is at the core of NPD and, moreover, that focusing on deficient empathy is the key for integrating three different, albeit structurally interrelated, levels of functional description, yielding descriptions as (1) a biological dysfunction, (2) an impairment of (intrapersonal) psychic functioning, and (3) as a form of maladjustment of (pro)social relatedness.

## The role of deficient empathy in pathological narcissism

The term “narcissist” colloquially (2) refers to the phenotype of self-absorbed, exploitative, egocentric, excessively demanding individuals with a strong tendency toward (predominantly: self-)idealization: they experience themselves as exceptional and grandiose and have little empathy for others. In the literature, articulations of this phenotype vary to some extent (3). However, there is significant overlap in the characteristics of a heighted sense of self and pronounced convictions of entitlement, and corresponding strategies of self-regulation. Assessing pathological narcissism requires not only a focus on intrapersonal functioning but on interpersonal functioning as well (4–6). Relevant for a differentiation of narcissistic modes of relatedness to oneself and to the world is the distinction between *grandiose* narcissism and *vulnerable* narcissism. In this paper, I focus on *pathological narcissism* which conceptually includes aspects of vulnerability and grandiosity as co-occurring symptoms. Both are not distinct traits, but manifestations of the same phenomenon, i.e., it depends on the more basic personality trait of intro- and extraversion whether either vulnerability or grandiosity is displayed (7). A narcissistic person's oscillation between grandiosity and vulnerability indicates a psychic disintegration on the intrapersonal functional level, which structurally correlates with maladaptive behavior on the level of social functioning. Vulnerability does not count as constitutive for the clinical diagnosis of pathological narcissism, since pathological narcissism is phenomenally not associated with low self-esteem. Nevertheless, vulnerability finds expression in a higher than average tendency of harming oneself (8), in suicide ideation (9), addictions (10), especially substance abuse [e.g., alcohol (11)]. This fact, together with a marked discrepancy between a positive future-orientation and an overall negative outlook (12) amounts to a an intrinsic vulnerability factor in NPD. The *vulnerable* phenotype of narcissism appears as introverted, hypersensitive, defensive, with a tendency for withdrawal and lowered self-esteem (13, 14). This is accompanied by increased ratings for anxiety and depression (15). Feelings of shame, inferiority and boredom are relevant symptoms of narcissistic depression as a special type of depression, distinct from ordinary depression (16).

Moreover, empirical findings suggest that threatening situations vary in their relevance for vulnerable narcissism and for grandiose narcissism. While grandiose narcissism is very sensitive to achievement setbacks, vulnerable narcissism is more sensitive experienced shame (17). Narcissism is diagnostically assessed either by means of a structural model (18) or by means of a spectrum model (19) and an assessment of empathy functioning is standardly included in understanding personality disorders in the *DSM-5* (20, 21) and the *ICD-11* (22, 23). While *intra*personal functioning is concerned with aspects of identity and the aspect of self-regulation, *inter*personal functioning addresses affective empathy, mind-reading, and also intimacy as central abilities for functioning well in interpersonal relations (24).

### The somatic description of deficient empathy in NPD

There is a staggering wealth of empirical data concerning the neurophysiological causes of mental dysfunction – e.g., causes of altered empathy in narcissism. From a methodological point of view, we have to note that some basic naturalistic underpinnings of empathy capacities have been identified. This fact illustrates how explanatorily strong the naturalistic paradigm has become. One can say without exaggeration that during the last decade, a “somatization” of personality disorders has taken place. This has become the conceptual background for the reassessment particularly of moral-psychological attempts to explain the role of empathy (in mental disorders). Neurophysiological explanations also put some alignment pressure on (social) psychological models that (still) operate with alternative notions of psychic functioning in order to explain psychic impairments in NPD “outside” of the naturalistic discourse of neurophysiological science. My core-deficit hypothesis is concerned with the *conceptual* analysis of NPD. The question is how to connect functional explanations framed in the naturalistic terms of the languages of neurophysiology to functional explanations framed in non-naturalistic terms of the languages of other disciplines, e.g., clinical psychology, social philosophy, hermeneutic social science. For a comprehensive theory of NPD the power of naturalistic explanations of cognitive and affective empathy are most welcome. However, we should not stop with functional descriptions at the biological and neurophysiological level of analysis. Rather, we should ask how such findings match with functional descriptions at other levels of analysis, e.g., clinical psychological descriptions of *psychic* impairment in persons with NPD. At this level of analysis, we understand empathy deficiencies in terms of maladaptive intersubjective practice. It is an interesting observation that naturalistic analyses often translate their empirical findings on deficient empathy in NPD into the language of *psychic* functioning, usually without caring at all about the proper conceptual clarification of notions like “impairment,” “inability,” “incapacity,” “distortion,” “deficit.”^2^ Typically, naturalistic analyses do not address the question whether we should *identify* neurophysiological events and processes with mental events and processes, or whether we should treat them as merely *correlated*. My impression is that the naturalistic studies that I have referenced resonate with the correlation paradigm (see Section Empathy deficit between *soma* and *psyche*).

From the neuroscientific point of view, empathic processes are grounded in dissociable neural systems (25). Empathy is conceptualized as the ability to affectively experience other persons' emotional states and as the ability to recognize and understand other persons' emotional states. A prerequisite for this is the ability to monitor oneself and to maintain and regulate self-other awareness (21) in order to differentiate between one's own and others' experiences (26). *Affective empathy* includes responsiveness to affectivity displayed by others, plus emotion-eliciting stimuli, which is not the same as the ability of mirroring others in one's responses (27). It is associated with (partially) distinct systems – all require activation of the superior temporal cortex – that show increased activation (amygdala, insula, ventrolateral prefrontal cortex), respectively, when agents respond to emotional expressions of others (28). Affective empathy develops ontogenetically earlier than cognitive empathy. Hence, from a social-philosophical perspective we can say that *re*cognition of the other “is ontogenetically (and conceptually) *prior*” to cognition [cf. (29), p. 354]. Empathic concern sets on during the second year of life, and its development depends on whether interactions either hinder or support empathic concern for others and/or support self-other-awareness (30, 31). Genetics (32), temperament and character (33) determine the development of empathic capacity in general. However, how specific genetic and environmental factors contribute to the development of personality disorders, and how genes particularly influence empathy deficiencies in narcissism, these issues continue to be controversially debated and remain a topic for future research (34, 35).

Person are normally capable of *perspectives-taking*. The development of this ability presupposes an imaginative faculty in order to attribute different emotions, attitudes, and desires to other persons in a given situation (36). Empathy is interpersonally trained and is consequential for psychosocial development (37, 38). Experimental research on empathy in narcissism indicates a stronger deficit in emotional empathy rather than in cognitive empathy, highlighting the factor of psychosocial development. A lack of intersubjective recognition, especially in terms of emotional neglect (39) and abuse, figure prominently in the literature. It is assumed that they constitute a pathogenic potential for the development of NPD (40, 41). *Nota bene*: (primary) narcissism is a normal aspect of children's development (42) and needs an age-appropriate satisfaction – a reflection of the grandiose self of the child – for a healthy psychic development. If such satisfaction is not forthcoming, i.e., when empathic reactions of the caregivers are missing, or when the child is overwhelmed by a caregiver's own grandiose self-expectations, according to interactional psychoanalysis (43) this constitutes a causal factor for developing pathological narcissism (44). The specific parental style, the inter-generational consequences of narcissistic relational styles, and the role of distorted self-other-awareness in families establishes a research field of its own (45, 46).

While affective empathy has a subcortical basis, *cognitive empathy* is associated with a network of cortical regions that enable mindreading-related neural processing (47). Empathic ability in the sense of *perspective taking* implies being able to attribute more complex states (thoughts, motives, intentions, etc.) to others, to change perspective and also to take an impartial point of view. This in turn requires overcoming egocentricity in perspective-taking in favor of empathizing with another person's situation and mental states. The dissociation between affective and cognitive empathy has been central for criticizing moral psychological approaches that solely rely on perspective-taking for explaining prosocial motivation. Empathy theories that do not take into account that the ability to take perspectives develops later than the ability to respond affectively to the *suffering* of others are open to empirical attack (48). Effective empathy goes, however, “beyond perspective-taking” as Jordan Carpenter and colleagues have suggested with the *mind-reading motivation model (MRM)* (49), according to which the individual differences of agents' willingness to get engaged in understanding the perspectives and mental states of other people is described. Conceptually this closes the gap between a mere registering another person's condition and being *actually* motivated in figuring out what others think, even if it is of no explicit personal relevance. Individuals that score low in MRM show a lower propensity of exposing themselves to others' perspectives. Exactly this is key to describe the *empathy deficit* in narcissism: it is a lack of *real* interest for the thoughts and feelings of others, and often rests on a decision for staying rather detached from others. Low affective empathy scores in pathological narcissism relate strong with grandiosity (50), and a lowered performance in perspective taking has been measured with respect to decision-making tasks (51). Individuals with NPD display also lower levels of perspective taking, when they have to respond in test-scenarios to questions that explicitly ask for *the motivation to become empathically* concerned in with others [cf. (52), p. 7]. These empirical results make a differentiation between a fundamental *lack* (53) and a reduced propensity to *recognize* the feelings and needs of others in NPD [cf. (19), p. 5]. This conceptual change of empathy incapacity has been also adopted to the fourth edition of the *DSM–IV* (54, 55). Individuals with NPD might be capable of processing affective information, but *decide* not to response empathically to others [cf. (52), p. 7], at least, when it is not directly beneficial for them to show concern in a specific situation, which also indicates that displaying empathy may become itself instrumentalized in NPD.

The empathy deficit then seems to be hybrid: albeit individuals with NPD can *register* the affective states of others, the ability to be emotionally *motivated* by them is insufficient and depends on the specific situational conditions. Considering the role of perspective-taking, individuals with NPD are apparently capable of imagining what moves other people, can infer how they might behave and how they themselves *should* behave. Narcissists are also capable of *appearing* compassionate or concerned. If narcissists can imitate (“fake”) these reactive attitudes of empathic concern they apparently have a sufficient cognitive understanding of the concept of empathy and/or compassion, and are also able to differentiate when the empathic responses technically should be exhibited. Empathy is (gradually) displayed as long as it severs own interests and can become instrumentalized to the detriment of others (56). The empirical studies are particularly helpful to assess the impairment of *moral* competence in individuals with NPD. Granted that higher-order cognitive processes (self-reflexivity) are necessary for moral competence (such as the understanding of action guiding maxims or higher-order volitions) it may be especially the insufficient affective motivation that explains the unwillingness of acting in conformity with norms (particularly harms-norms) in NPD. In this respect, the capacity for moral judgment should be further explored according to this bias of cognitive and affective functioning in NPD.

### The psychodynamic description of deficient empathy in NPD

Psychodynamic studies on pathological narcissism resonate with the empirical findings, and ever since have stressed the oscillation between the vulnerable and grandiose aspects as main characteristic of pathological narcissism. In contrast, this has been a rather neglected aspect in the diagnostic manuals and the DSM-4 NPD category has been largely criticized for focusing mostly on overt grandiosity and less on the vulnerability dimension (57, 58). It is particularly with respect to psychodynamic approaches that the interrelation of grandiosity and vulnerability becomes explained in terms of psychic functioning and that the narcissistic vulnerability is addressed as potentially increasing the tendencies to devaluate and to “act out” onto others in NPD. The crucial symptom of narcissistic personalities is pronounced feelings of insufficiency and these feelings are compensated with fantasies of omnipotence and greatness. Persons with NPD – albeit being relatively unreliable in their empathic responses to others – are themselves highly dependent on how others see them due to their fears of social rejection and worries about threats of their social status (59).

What a psychoanalytic view on NPD exemplarily stresses is the reinterpretations of reality, which particularly manifest in misperceptions of both, the environment and own possibilities [cf. (60), 201ff; transl. KJA]. Erich Fromm – who frees himself from Freud's conceptualization of narcissism within the constricting frame of reference of the libido theory [(61), p. 37–74, 65ff.], accordingly defines narcissism as

“[…] an orientation in which all one's interest and passion are directed to one's own person: one's body, mind, feelings, interests, and so forth. (…) For the narcissistic person, only he and what concerns him are fully real; what is outside, what concerns others, is real only in a superficial sense of perception; that is to say, it is real for one's senses and for one's intellect. But *it is not real in a deeper sense, for our feeling or understanding*. He is, in fact, aware only of what is outside, inasmuch as it affects him. Hence, he has no love, no compassion, no rational, objective judgment. The narcissistic person has built an invisible wall around himself. He is everything, the world is nothing. Or rather: He is the world.” [(62), p. 117].

Otto Kernberg who sees pathological narcissism proportionally increasing to the level of aggression mentions that the grandiose self of narcissists is a construct of all the positive and idealized characteristics of themselves and also of others into an unrealistic self-image. Devaluations of this image are split off or projected onto others (63). These psychic defense mechanisms, which serve for self-regulation and maintenance of the grandiose self-image, may be one reason why the grandiose type scores higher in life-satisfaction (64), as criticism of others is not perceived as a signal for self-assessment. On the contrary, a decline in self-esteem due to negative feedback (65), a record of unstable, superficial relationships (66), risk-taking and impulsive behaviors that significantly affect health (67) reveal the not so “happy face” of narcissism (68). With a closer look on empathy distortions as maladjustment, the *dissocial* tendencies associated with NPD have to be mentioned: Others are intentionally harmed in particular, if they are perceived as threatening, because they are scratching the self-ideal that determines the self-image of individuals with NPD. This is a reason for why NPD comes along with a relatively poor compliance to treatment, and what makes NPD one of the most difficult conditions to treat (69, 70) – for instance with respect to processes of countertransference (71) – and which often requires an adjustment of therapy and/or special treatment techniques for NPD (72).

The psychoanalyst Udo Rauchfleisch (60) elaborates the oscillation between the grandiose and vulnerable dimension in close relation to *dissocial behavior* in a variety of psychopathological conditions. His analysis is consistent with the previous mentioned motivational lack of empathic concern in NPD. His analysis allows to focus on the intrapsychic constellation in NPD as characterized by an “oppressive dictate of a hypertrophied ego ideal,” which demands the narcissist is unable to cope with. As a libido-economic consequence resulting feelings of insufficiency are concealed, which is often accompanied by a “diffuse anxiety” and “dysphoria,” and compensated for by impulsive actions [cf. (60), p. 201 ff.]. NPD consequently often includes harming others inasmuch as narcissistic agents engage in interpersonally exploitative behavior (often addressed as narcissistic rage) *because* they have these unrealistic expectations and hypertrophic demands that express in particular claims for loyalty, support, and admiration from others. The narcissistic maladaptive interpersonal functioning structurally corresponds with a “superego pathology (73)” on the intrapersonal functional level of psychic organization. Even if narcissists do not suffer from a maldevelopment of the super-ego as such, they exhibit an *integrational deficit* of the super-ego demands. If we apply this analysis [(60), p. 77ff] of the dissocial personality organization to NPD, the peculiarity of pathological narcissists is that their defense mechanisms are not mainly directed against aggressive and libidinous impulses of the id, but rather become directed against certain parts of the superego instance itself. The mechanisms of projection and projective identification then play a decisive role: In the projection of the superego demands, for example, onto other individuals or external authorities, externalizing and splitting tendencies – e.g., the split between the exaggerated ego ideal and a negative self-representation “from within,” and that between the “totally good” and “totally bad” objects “outside” – can be maintained. In parallel, the second mechanism of identification (with a superego carrier) prevents the internalization of the conflict-ridden demands or a realistic self-assessment: Albeit the superego demands (e.g., the knowing that one should respond to the needs of others, act in accordance with norms that prohibit harming others, etc.) remain “outside,” these stay nevertheless effective inasmuch as they reappear as threats represented by other persons or institutions (74). As a consequence, narcissists tend to depreciate themselves (vulnerability) or reactivate the greater self (grandiosity). This leads not only to a stance of arrogance, or mere indifference, but to aggressive or otherwise dissocial behavior that is (overtly/covered) displayed toward others, in this case: particularly toward superego carriers.^3^

The psychodynamic explanation contributes to a deeper understanding of NPD as it resonates with the latest empirical results on the interrelatedness of grandiosity and vulnerability in NPD, and, moreover it conceptually specifies psychic functioning on the meso-level of description of the empathy deficiency: NPD involves a pathological superego constellation – a psychic disintegration – according to which the unemphatic behavior is causally explained within the framework of an alternative model of psychic functioning. This highlights the explanatory power of non-reductionist accounts of mental processing on the one hand, but simultaneously allows for an interdisciplinary integration, as the psychodynamic model stays conceptually open for being empirically further “grounded” in and/or testified against other functional descriptions of empathy distortions provided by neuroscientific explanations of empathy deficiencies, on the other hand. The latter, in contrast, rather tend to be reductionist in their explanations of empathy processing in NPD in terms of biological functioning, but this does not rule out an alternative understanding of the pathogen dynamics of psychic disintegration on a different explanatory level of empathy deficiencies in NPD. Moreover, psychodynamic approaches bring with them the surplus of an explicit *psychosomatic* understanding of pathology, although the liaison between *soma* and *psyche* has been a complicated one for the psychoanalytic discipline, too (75). Nota bene: For the purpose of my analysis it is not needed that the dynamics of the interplay of the psychic instances have to become one-to-one (re-)translated into the “neuroanatomical” language; but what is, indeed, important for an integrative perspective on the empathy deficit in NPD, is to understand the intra– and interpersonal sphere as *conceptually* inextricably intertwined functional units of the psychic apparatus. From a phenomenological perspective it is clear that empathy distortions affect both, intra- and interpersonal functioning, i.e., the self-*and*-world–relatedness of narcissistic individuals. This is conceptually grasped with the psychodynamic description of impaired psychic functioning. Consequently, *psychic* disintegration on the intrapersonal level conceptually relates to *social* disintegration described on the level of interpersonal functioning.

### The philosophical description of deficient empathy in NPD

A third class of functional descriptions of the empathy deficit is provided by social-philosophical view, according to which NPD expresses as a distortion of the intersubjective practice of recognition (76). Empathy enables us to develop a relatively stable stance of an interested involvement with others: It provides an open space of experiential possibilities of relatedness. The philosopher Axel Honneth describes this as primordial to all kinds of objectifying modes of self-and-world–disclosure (77). In his discussion of Lukács' concept of reification he makes a crucial assumption that is not only correct from a (brain-)developmentally perspective, but also true with regard to the sphere of intersubjective action in general: namely, that we – as the relational beings that we are – are always already affectively attuned to this world and are engaged in modes of interested participation in relation with others. This is the opposite to an objectifying mode of self-and-world–disclosure.^4^ The empathy deficit of individuals with NPD reveals in the tendency for a (pre-)intentional detachment, an inability to genuinely engage with others (as opposed to merely feigning concern for others in order to appear “social”). This is the socially impairing aspect of the empathy deficit in narcissism. If qualitative relations to others have a priority over objectifying relations to them, then the empathy deficit hinders an interested involvement with others in terms of (inter-)subjective recognition. The narcissists' empathy deficit implies the “active” forgetting of this priority (of the other): even if they register the needs of others, their pathological self-centeredness restricts the experiential possibility for empathic concern for others. Quite an opposite view on others comes with this particular mode of active forgetfulness of recognition: The stance of reifying others and to perceive them as mere means to an end. In NPD this can include also modes of “false” recognition (e.g., when others are not recognized for what the truly are, but become “reduced” to a certain function or property that can be valued for a certain purpose). When reification forms the rigid habitual pattern for “relatedness,” this enables the forms of *mal*adjustment, that in its typical forms of narcissistic violence shows one significant feature: namely the exploitation and abuse of others (78–80). This is the explicit *dissocial* aspect of severe forms of pathological narcissism. The empathy deficit reveals as a lack of or as false recognition, which practically demolishes what we normally are taking for granted in our relations to others. In one's daily encounters one is repeatedly confronted with conflicting emotions and commitments, and central to psychic health is the flexibility to cope with these in certain situations. The pathological core of narcissism consist in often not being able to literally feel and to adequately assess these situations as conflicting at all, and/or to readjust in practice, respectively. NPD then comes along with a significant restriction of experiential possibilities for *pro*-social relatedness, for *being-with* others. This can, of course, cause problematic social interactional patters that are revealing the vulnerability of the interaction partners: one the one hand, people with NPD might feel misunderstood, themselves not appreciated, suspect others to be ill-willed, etc., while their interaction partners are disturbed by their self-centeredness and lack of awareness for the feelings and real needs of others, on the other. If NPD is not seen for what it is – a pathological condition that has socially impairing dimensions and yields a high vulnerability – this might foster stigmatization of patients with NPD.

As an interim conclusion it can be stated: The coherence of an integrative model of the empathy-deficit in NPD requires the structural consistency of the respective different functional explanations provided at the distinct descriptive levels (top down/bottom up). Insofar as the functional descriptions of the affective motivational deficit on the micro level (Section The somatic description of deficient empathy in NPD) is structurally consistent with the analysis of psychic disintegration on the meso-level (Section The psychodynamic description of deficient empathy in NPD), and this is consistent with the description of the empathy deficit as lack of recognition on the macro level (Section The philosophical description of deficient empathy in NPD) my analysis exemplarily shows the compatibility of different functionalistic descriptions within an integrative model of empathy deficit in NPD. In the following section some methodological considerations finally are addressed.

## Discussion: The core-deficit-hypothesis

### Empathy deficit between *soma* and *psyche*

The biological approach to narcissism promises a concretization and naturalistic foundation by guaranteeing empirical objectivity, but as a result, might cast doubt on the notion of narcissism as a *mental* disorder. Reductive positions are therefore often associated with a “disqualification” of alternative explanations of mental processes, especially when these are seen as completely reducible to (or even “identifiable” with) physical processes. Instead of abolishing the conceptualization of narcissism as *mental* disorder, it seems much more reasonable to assume a correlation between mental processes and physiological processes *even if* the scientific convincingness of neurophysiological explanatory models might be already considered as providing some grounds for rejecting alternative models as equally reasonable for a conceptualization of mental (dys-)functioning in narcissism. Methodologically it is nevertheless still justified to speak of NPD in terms of *psychic* impairment, even if the interest in an fully objectively accessible “localization” of the narcissistic mind, e.g., in brain-organic explanatory models, would have been already fully satisfied. There have been, indeed, several neurophysiological foundations of pathological narcissism suggested (52, 81–83), as has been proposed also on a larger scale for other types [e.g., for the antisocial personality disorder (APD), or Borderline Personality Disorder (BPD) (84)] of the cluster B-personality disorders (85) with respect to altered empathy processing. Consequently, the clinical studies allow one to trace the characteristics of narcissist's manifold “relational” problems back to significant changes of predominantly affective empathy [lesser to cognitive empathy (5)].

My analysis stresses the *conceptual* distinction of different descriptive levels of “empathy.” What reductionist approaches conceptually often fail to address is that *psychic functioning* cannot be fully deciphered solely in naturalistic terms – even if one *can* describe mental processing with respect to the *factum brutum* of empirical data provided by brain scans, salvia and blood samples, skin conduction and blood pressure tests, etc.^5^ The notion of ‘psychic functioning' is, however, neither to be equated with the notion of ‘mental processing', nor with the notion of ‘physiological functioning', but rather mediates between both levels of descriptions, and therein serves as an independent category for describing empathy deficiencies in NPD. If this is the case, neither the parlance of the “mental” is fundamentally ruled out with my three-level analysis, nor does the integration of a reductionist view inevitably lead to a relapse in some sort of “brain-mythology” when we speak of psychic (dys-)functioning. Moreover, a major distinction, namely between *explaining* and *understanding* (86, 87) should generally be kept in mind: The narcissistic brain is something to be explained, but the narcissistic mind is something we have to understand. Naturalistic views on narcissism literally allow to “emphasizes” a core deficit as a *pathology*, but an understanding of it – the *meaning* of empathy impairment – in NPD is provided by an evaluation against the normative backdrop of theories of psychic health, wellbeing, and (pro-)social relatedness. The latter keeps the phenomenal reality of NPD “in mind” from a live-worldly view, without forgetting the former “scientific project” of explaining narcissism in somatic terms within a naturalistic paradigm. As such, reductionist analyses are inevitable useful to objectify certain somatic changes in empathy responses and therein have the explanatory power for additionally empirically “backing” non-reductionist explanations of psychic impairments in NPD. What can be measured is at least neurophysiological reactions [e.g., stress responses (88)] to specific social situations, while it is due to the dynamics of re-enaction that these somatic changes manifest as (rigid) evaluative pattern for self-and-world–disclosure. This already implies an conceptual understanding of the *pathological situatedness* of NPD as irreducible to neurophysiological dynamics, but as ideally well-informed by them (89).

### The empathy deficit between conceptual over-complexity and under-complexity

A second methodological doubt might arise with respect to either a *complexity-reduction* or, on the contrary, an *over-complexity* with the focus on empathy as the core deficit of NPD. Generally it is to be assumed that it is a variety of physiological mechanisms that determine the expression of different subtypes and degrees of abilities in and for personality disorders such as narcissism [(52), p. 2]. Moreover, it might be also especially the “plus” of comorbidity that explains *how* empathy-related impairments *exactly* realize in their particular forms in narcissism, and, moreover, in distinct types of Cluster-B-personality disorders. Skeptics could respectively label the core-deficit hypothesis as “naïvely” under-complex or over-complex, thus insufficient to address empathy as the conceptual core of NPD (or to even account for a “core” at all). Starting with the latter, it is obvious that empathy-(cor)related neurophysiological dysfunctions actually provide a very (if not *the* most) promising account for a specification of narcissism as a *disorder* (90–93) – we can objectify the *dys*- of function – and, as such, puts other explanatory models, which rely solely on evaluative criteria in their place. The core-empathy-deficit covers a large number of key symptoms of NPD. Not only the singular functional impairments of affective and cognitive empathy as such, but also the discrepancy of different levels of this functional units together determine the core of NPD.

Secondly, in lights of the different functional roles empathy has not only for the explanation of NPD, but also for a range of other clinical conditions, this is not an objection against, but rather an argument for empathy as the core-deficit in NPD, because exactly this is the starting point for a more-fine-grained specification (e.g., on the molecular-biological level) for determining the *distinct* impact of empathy for NPD, and explicitly in comparison to other disorders. The core can be conceptually defined according to a differentiation of particular functional *patterns* of empathy-related dysfunctions for and within narcissism as *distinct* disorder type. In narcissism we have the interesting combination of relatively intact cognitive together with impaired affective empathy, and this pattern can be continued to be refined in comparison to other disorder types (such as APD, BPD). This might reduce conceptual over-complexity (here: exemplarily) for the descriptions of the empathy deficit in terms of biological dysfunctions.

Thirdly, under-complexity can be conceptually reduced with respect to the potential of the core-deficit to integrate different levels of description and disciplinary views (neurophysiological, psychological, sociological, philosophical, etc.) that respectively specify the meaning of the empathy deficiencies in NPD due to an interdisciplinary research objective. In order to obtain the conceptual consistency of the three-level analysis, a structural requirement is relying on functionalism (teleological, etiological, system-functional, and propensity functional descriptions of empathy). The explanations provided by different functional explanations then can become approved (testified against each other) with respect to their structural (in-)consistency for each and among different levels of description in my analysis. The higher the consistency, the more coherent is the particular explanatory structure (or pattern) of functional descriptions for empathy distortions in NPD. The basic structural integration must include in my analysis (top down/bottom-up) a reference to (1) biological dysfunctions, (2) intrapersonal impairments of psychic functioning, and (3) a distortion of social relatedness as it has been exemplarily sketched here.

### Empathy deficit between mental disorder and social pathology

Finally, one could stipulate that the “personality” of a person is basically nothing that can be addressed in reductionist terms, or should be object of any medical assessment or diagnosis (even if we could objectify the underlying somatic dynamics), because it falls within the protective sphere of privacy, agency and personhood. Such positions could be carried out under the auspices of an (allegedly) pathologizing of narcissistic character traits, and/or even of a “moralization of diagnostics”, especially when the “harmful” dimension of interpersonal difficulties of narcissism are highlighted with reference to empathy deficiencies. If a normative standard – for instance, norms of prosocial motivation – mutates into a clinically diagnostic yardstick for assessing individuals, this might be untenable from a scientific, (allegedly) value neutral point of view. Reminding on the debate about whether to keep narcissism as a clinical disorder category (94), some skeptics might consider narcissism as mere character “accentuation,” which is – however impairing or otherwise harmfully experienced these traits might be – no reason to suspect a mental disorder; especially not, when related behavioral styles are widely common, or even get promoted for their adaptive potential in certain fields of social practice (95, 96). In the context of methodological considerations of the psychiatric classification systems it can be explicitly pointed out that conflicts between society and the individual alone do not provide a sufficient basis for the attribution of a mental disorder. The classification manuals follow here an important intuition, which also owes itself to a confrontation with psychiatry-skeptical positions, when it is stated in the general definition of mental disorders:

“Whatever its original cause, it must currently be considered a manifestation of a behavioral, psychological, or biological dysfunction in a person. Neither deviant behavior (e.g., political, religious, or sexual) nor conflicts that are primarily between the individual and society are mental disorders unless the deviance or conflict is a symptom of a dysfunction in the individual.” [APA. DSM-IV-TR. (96). p. xxi-xxii.]

Although this theoretical limitation is intended to avoid defining mental disorder solely in terms of social deviance, it does not guarantee that misdiagnoses can always be avoided. These conflicts cannot, in my opinion, sufficiently justify a clinical diagnosis, but are admittedly important indications for a *differential* diagnosis in clinical practice. Moreover, the fact that narcissistic traits are apparently so widespread that they might even have become some standard social norm does not necessarily imply, that a pathology can be fundamentally ruled out (97). Exactly the opposite would have to be assumed, if one takes seriously, for example, studies on *social pathologies* (98, 99). With the psychoanalysts' and sociologists' Erich Fromm analysis on the *anatomy of human destructiveness* (100) one could state, that any trying to “normalize” NPD rather would indicate that something can be fundamentally wrong in such a society (or with certain institutions), as it fails to recognize the “pathology of normalcy” (101). At least this is the case, when there is an systemic (even institutionalized) indifference toward, or even a denial of “the intentional use of physical force or power, threatened or actual, against oneself, another person, or against a group or community, that either results in or has a high likelihood of resulting in injury, death, psychological harm, maldevelopment or deprivation” at play [(102), p. 1084, 104]. From a social-cultural diagnostic view provided by Critical Theory such strategies of normalization – in analogy to strategies of pathologizing – could both even be reframed as a signifier of a *second-order social pathology* [(29), p. 347] inasmuch as the second-order sense of an allegedly obviousness of first-order beliefs or normative assessments may contribute to the perpetuation of practice forms that are the relevant causal factors for reproducing these beliefs and assessments (e.g., the power of psychiatric diagnostic politics to declare certain phenomena as (non)-pathological). In severe forms of NPD the condition involves intentional harming of others, and altered empathy certainly contributes to it [excluded here the harm done by empathy induced *altruism* (103, 104)]. A diagnostic view on narcissism that frees itself from the assessment of the complex harm-dimension of NPD denies not only its clinical phenomenality from a live-worldly perspective, but also seems to ignore a scientific understanding of narcissism as *disorder* of interpersonal functioning, for instance, when the association between narcissism and aggression that has been empirically supported in adults and adolescents is denied, or when the particular meaning of harm as referring to individual suffering from vulnerability in NPD is not fully recognized (105). Nota bene: An assessment of actions is logically always different and has to be carefully discriminated from the assessment of personality from an objective clinical diagnosis, but non-trivial self-and other harming actions and behavioral styles must be at least reconsidered as corelated to empathy distortions in pathological narcissism. Considering non-trivial other-harming of additional diagnostic relevance for the diagnostics of NPD appears at least plausible with the focus on an empathy deficit as a causal factor for violence in narcissism, particularly when exactly this simultaneously can be understood in relation to narcissistic vulnerability, i.e., as an expression of social maladjustment due to an altered scope of experiential possibilities to empathically engage with others.

## Conclusion

I have examined NPD from an conceptual perspective and focused on its core: the empathy-deficit. This has been reconceptualized with an integrational model that relates different functional descriptions provided by three structurally interrelated descriptive levels: The micro-level of biological dysfunctions, the meso-level of psychic impairment, and the macro-level of distortions of intersubjective practice, that together shape the interdisciplinary view on NPD in this analysis. Although my analysis is restricted in scope, I hope that I have provided some reasons to accept that an integrative approach toward empathy, which stresses on the ‘psyche' as a mediating category, allows to bridge some trenches between the naturalistic explanation and normative understanding of empathy deficiencies in NPD.

## Author contributions

KAJ is the contributing and corresponding author of this article.

## Funding

This work was funded by the Center for Human Nature, Artificial Intelligence, and Neuroscience (CHAIN).

## Conflict of interest

The author declares that the research was conducted in the absence of any commercial or financial relationships that could be construed as a potential conflict of interest.

## Publisher's note

All claims expressed in this article are solely those of the authors and do not necessarily represent those of their affiliated organizations, or those of the publisher, the editors and the reviewers. Any product that may be evaluated in this article, or claim that may be made by its manufacturer, is not guaranteed or endorsed by the publisher.

## Footnotes

^1^This is paradigmatically described in the case review of “Jane” in the study by Bonney Reed-Knight and Sarah Fischer. The patient explains this particular phenomenon by saying: “If they're not meeting my needs, they're in my way, if they're meeting my needs, I need them.” [(1), p.470].

^2^The notion of “deficit” suggest a gradual theory of incapacity. While “defects” are structurally manifested, irreversible deficits – biostatistical measurable dysfunctions that can no longer be compensated by other functions (functional networks, modules) – deficits can be captured as reversible and structurally (at least partially) compensable dysfunctions in a network or module of functions. Analogously, these terms are obviously also used on the level of description of abilities. *Incapacity*, as irreversible inability, mark the extreme form of impairment, while deficits are characterized by a changeability in both directions of improvement and deterioration in a corresponding area of ability. Within the field of psychic impairments an application of the gradual view indicates to specify the degree of dysfunctionality and that of inabilities in equal measure. Moreover, NPD is apparently also characterized by a certain degree of functioning, which can also be taken into account by a gradual view provided with the notion of deficit. Another term is that of *distortion*, which is referred here to explain gradual forms of social *maladjustment*, while maladjustment implies an assessment against a normative theory of social behavior and wellbeing.

^3^“The adherence to the >>evil < < partial object, which appears again and again as a punishing superego in the outside world, becomes a necessity for the dissocial person, so that he can at least in this way maintain a certain self-identity.” Cf. (74), p. 82.

^4^Honneth does not rule out that an objectifying stance is begin if it is conducted in a normative permissible manner. What he is targeting with his analysis, is a “forgetfulness of recognition” that happens in reification, and that has the potential to erode the very preconditions for an intersubjective practice based on respect and understanding, thus for an ethical form of social life: Honneth writes “[…]this kind of “forgetfulness of recognition ”can now be termed “reification.” I thereby mean to indicate the process by which we lose the consciousness of the degree to which we owe our knowledge and cognition of other persons to an antecedent stance of empathetic engagement and recognition.” cf. (77). p. 52–63, 56.

^5^For a systematic overview see: (82), p. 8–11 (Table 2).
